# Surface Phosphorus-Modified Mo_2_C MXene Toward Boosted Oxygen Reduction Reaction for Mg–Air Battery

**DOI:** 10.3390/nano16211384

**Published:** 2026-10-28

**Authors:** Canbo Hu, Xinyue Gao, Guoqin Cao, Fanfan Liu

**Affiliations:** 1 State Center for International Cooperation on Designer Low-Carbon & Environmental Materials (CDLCEM), School of Materials Science and Engineering, Zhengzhou University, Zhengzhou 450001, China; hucanbo@stu.zzu.edu.cn (C.H.); gxy01@gs.zzu.edu.cn (X.G.); 2 National Key Laboratory of Special Rare Metal Materials, Zhengzhou University, Zhengzhou 450001, China

**Keywords:** Mg–air battery, Mo_2_C MXene, oxygen reduction reaction, phosphidation, surface engineering

## Abstract

Mg–air batteries have garnered substantial research attention owing to their high specific capacity, low electrode potential, environmental benignity, and abundant natural magnesium reserves. Nevertheless, sluggish oxygen reduction reaction (ORR) kinetics at the air cathode severely restrict their practical implementation, which necessitates the development of low-cost, high-activity non-precious metal electrocatalysts. Herein, modified Mo_2_C@P electrocatalysts are successfully synthesized via phosphidation of Mo_2_C mixed with sodium hypophosphite (NaH_2_PO_2_) under thermal treatment. Multiple characterization techniques are employed to systematically investigate the crystal structure, chemical bonding configuration, micromorphology, and ORR electrocatalytic performance of the as-prepared materials. Meanwhile, Mg–air battery evaluations are carried out in 3.5 wt.% aqueous NaCl electrolyte. The results reveal that the Mo_2_C MXene backbone is well retained. Phosphorus doping is confined to surface modification and is accompanied by the formation of P–O and P–C bonds. The stacked lamellar architecture undergoes surface reconstruction, generating a uniform particulate morphology. The ORR electrocatalytic activity exhibits a volcano-type trend with phosphidation temperature, and Mo_2_C@P-350 achieves the highest limiting diffusion current density of 2.87 mA cm^−2^. The Mg–air battery assembled with Mo_2_C@P-350 delivers outstanding and stable discharge voltages of 1.19 V at 5 mA cm^−2^ and 1.13 V at 10 mA cm^−2^, along with optimal rate capability. This work demonstrates that mild-temperature phosphidation represents a reliable surface-engineering strategy for constructing high-performance ORR electrocatalysts.

## 1. Introduction

In recent years, the ever-increasing demand for clean and sustainable energy has stimulated intensive research on advanced electrochemical energy storage and conversion systems [1,2]. Among various candidates, metal–air batteries have attracted considerable attention because they directly utilize atmospheric oxygen as the cathode reactant, which endows them with theoretical energy densities far exceeding those of conventional lithium-ion batteries [1]. Within this family, magnesium–air (Mg–air) batteries are regarded as one of the most promising next-generation power sources owing to the unique merits of the Mg anode, including a high theoretical specific energy of 6.8 kW h kg^−1^, a theoretical cell voltage of 3.1 V, a large volumetric capacity (3832 mA h cm^−3^, nearly twice that of lithium), natural abundance, low cost, and an intrinsic resistance to dendrite formation [3,4,5]. Nevertheless, the practical performance of Mg–air batteries remains far below these theoretical values. In addition to anode-side issues such as self-corrosion and passivation by insulating discharge products, the sluggish kinetics of the oxygen reduction reaction (ORR) at the air cathode is widely recognized as the principal bottleneck limiting the discharge voltage, power density, and energy efficiency of Mg–air batteries [5,6]. Therefore, the rational design of low-cost, highly active, and durable ORR cathode catalysts is of central importance for advancing Mg–air battery technology.

Pt-based materials have long served as the benchmark ORR catalysts, but their scarcity, high cost, and insufficient durability have motivated the exploration of non-precious alternatives [7,8]. For example, α-MnO_2_ nanorods supported on three-dimensional graphene delivered a half-wave potential of 0.81 V and a peak power density of 106.2 mW cm^−2^ in Mg–air fuel cells [9], and atomic Fe–N_x_ coupled open-mesoporous carbon nanofibers were demonstrated as efficient oxygen electrodes for Mg–air batteries [10]. More recently, two-dimensional transition-metal carbides and nitrides (MXenes), first obtained by the selective etching of Ti_3_AlC_2_ in 2011, have emerged as a versatile platform for electrocatalysis because of their metallic conductivity, hydrophilic surfaces, and tunable surface terminations [11,12,13,14]. Among the MXene family, molybdenum carbide (Mo_2_C) MXene, synthesized by the exfoliation of Mo_2_Ga_2_C, combines a Pt-like d-band electronic structure with high electrical conductivity and has thus been considered a promising ORR electrocatalyst [15]. Theoretical calculations predicted that Mo_2_C MXene can serve as an excellent substrate for ORR-active overlayers [16]; Fe single atoms anchored on Mo_2_CT_x_ exhibited high activity toward selective oxygen reduction [17]; and, notably, Mo_2_C MXene was employed as the air cathode of a biodegradable Mg–air battery, in which it suppressed the competing hydrogen evolution reaction and enabled an operating voltage of 1.4 V [18]. Despite this promise, Mo_2_C MXene still suffers from a limited density of intrinsic active sites, the restacking of nanosheets, and, in particular, poor oxidation stability under oxidative ORR conditions, which can induce in situ transformation of the carbide phase and degrade the catalytic performance [17,19,20]. These drawbacks highlight the necessity of tailoring the surface chemistry and electronic structure of Mo_2_C MXene to simultaneously enhance its activity and stability.

Heteroatom modification, especially phosphorus incorporation, provides an effective route to regulate the electronic structure of transition-metal carbides, because P doping can shift the d-band center, optimize the adsorption energetics of oxygenated intermediates, and create additional P–C/P–O active configurations [21,22]. In this work, phosphorus-modified Mo_2_C MXene (Mo_2_C@P) catalysts were prepared via a facile temperature-programmed phosphidation route using sodium hypophosphite as the phosphorus source, and the influence of the phosphidation temperature on the crystal structure, morphology, surface electronic structure, and electrocatalytic behavior was systematically investigated. XRD, Raman, FTIR, SEM, and XPS analyses reveal that the layered MXene framework is preserved after phosphidation and that P is successfully incorporated in the form of P–C and P–O bonds. Benefiting from the optimized electronic structure, Mo_2_C@P phosphidized at 350 °C delivers the best ORR performance among the series in alkaline media, with a half-wave potential of 0.62 V and the highest limiting diffusion current density of 2.87 mA cm^−2^, outperforming pristine Mo_2_C (0.59 V and 2.24 mA cm^−2^). When assembled as the catalytic air cathode of a Mg–air battery, Mo_2_C@P–350 enables an open-circuit voltage of approximately 1.5 V and stable galvanostatic discharge at 5 and 10 mA cm^−2^, demonstrating that temperature-programmed phosphidation is an effective strategy for developing high-performance MXene-based cathode catalysts for Mg–air batteries.

## 2. Materials and Methods

### 2.1. Preparation of Mo_2_C@P Catalytic Materials

Phosphorus-modified molybdenum carbide (Mo_2_C@P) catalysts were prepared by mechanical mixing followed by temperature–programmed phosphidation. Specifically, Mo_2_C powder and sodium hypophosphite (NaH_2_PO_2_) were weighed at a mass ratio of 10:1 (approximately 55 mg in total) and ground thoroughly in an agate mortar to achieve homogeneous mixing of the two phases. The mixture was then transferred into a tube furnace and purged with nitrogen for 1 h. After purging, the furnace was heated to 250, 350, and 450 °C at a rate of 5 °C min^−1^ under a nitrogen atmosphere and held at each temperature for 2 h to perform the phosphidation treatment. The samples were then allowed to cool naturally to room temperature in the furnace, yielding Mo_2_C@P powders phosphidized at different temperatures, which are denoted as Mo_2_C@P–250, Mo_2_C@P–350, and Mo_2_C@P–450, respectively. The pristine Mo_2_C without phosphidation treatment was used as the control sample and is denoted as Mo_2_C. The underlying mechanism of the above phosphidation process is as follows: NaH_2_PO_2_ decomposes upon heating to release phosphorus-containing active species, which react with the surface and near-surface layers of Mo_2_C, introducing P–O and P–C bonds into the material and thereby achieving surface phosphidation modification of Mo_2_C. The phosphidation temperature is a key parameter governing the extent of phosphorus incorporation and the surface structure of the material: an excessively low temperature leads to insufficient phosphidation, whereas an excessively high temperature may lead to the thermal desorption of the phosphorus-containing surface species. Therefore, the temperature range of 250–450 °C was selected in this work to investigate the influence of phosphidation temperature on the structure and performance of the materials.

### 2.2. Preparations for the Assembly of Mg-Air Battery

The catalyst-modified air cathode was fabricated through acid treatment and blade-coating. Carbon cloth was cut into rectangular pieces of 7 cm × 3 cm, immersed in concentrated nitric acid for 10 h for acid treatment, then rinsed repeatedly with anhydrous ethanol until neutral and dried for later use. The catalyst slurry was prepared by mixing Mo_2_C or Mo_2_C@P, Ketjenblack, and polyvinylidene fluoride (PVDF) at a mass ratio of 7:2:1. The mixture was ground in an agate mortar for more than 30 min, followed by the addition of an appropriate amount of N-methyl-2-pyrrolidone (NMP) solution under thorough stirring to form a paste-like slurry. The resulting slurry was uniformly blade–coated onto the carbon cloth and dried in a vacuum oven for 10 h to yield the air cathode of the battery. For the Mg anode, a magnesium sheet with a length of 7 cm, a width of 3 cm, and a thickness of 0.5 mm was polished on both sides to remove the surface oxide layer, then rinsed with anhydrous ethanol and blown dry for use as the anode. The battery was assembled with 3.5 wt.% NaCl aqueous solution as the electrolyte, in which the air cathode, Mg anode, and electrolyte were integrated into a liquid-electrolyte Mg–air battery. The electrolyte concentration was chosen following the established practice in the Mg–air battery literature: 3.5 wt.% NaCl mirrors the average salinity of seawater, and concentrations of this magnitude are the most frequently employed in the reported aqueous Mg–air batteries, so that the performance obtained here can be directly compared with the literature data [23]. Compared with strongly alkaline electrolytes, in which the abundant OH^−^ captures the Mg^2+^ near the anode and promotes the accumulation of a dense Mg(OH)_2_ passivation layer that raises the anode dissolution potential and lowers the discharge voltage, the neutral NaCl electrolyte avoids such anodic passivation [5,6]. NaCl additionally provides satisfactory electrical conductivity, good chemical and electrochemical stability, low cost, and environmental benignity [23]. The custom-made mold had a height of 6.0 cm, a width of 7.0 cm, and a capacity of 12.0 mL, with an effective exposed area of 1.0 cm^2^ for both the cathode and the anode.

### 2.3. Material Characterization

The phase composition and crystal structure of the samples were analyzed by X-ray diffraction (XRD, Rigaku Ultima IV system) using Cu Kα radiation (λ = 0.154 nm). The diffraction patterns were recorded in the 2θ range of 5–70° at a scanning rate of 10° min^−1^. The functional groups of the samples were analyzed by Fourier-transform infrared spectroscopy (FTIR, Thermo Scientific Nicolet iS20 spectrometer). The samples were prepared by the KBr pellet method, and spectra were recorded in the range of 4000–400 cm^−1^. The structural characteristics of the materials were analyzed by Raman spectroscopy (Bruker Senterra) with a laser wavelength of 532 nm, and the spectra were collected in the range of 50–2000 cm^−1^. Scanning electron microscopy (SEM, ZEISS SIGMA) was employed to examine the morphology and microstructure of the samples. The surface elemental composition and chemical valence states of the samples were analyzed by X-ray photoelectron spectroscopy (XPS, ThermoFischer ESCALAB 250Xi spectrometer) with a monochromatic Al Kα radiation source (hν = 1486.6 eV), and all binding energies were referenced to the C 1s peak at 284.8 eV.

### 2.4. Electrochemical Measurements

The oxygen reduction reaction (ORR) electrochemical performance tests of the catalysts in this work were all conducted on a CHI760E electrochemical workstation(AMTECT Company) at room temperature using a three-electrode system. An Hg/HgO electrode served as the reference electrode, a carbon rod as the counter electrode, a catalyst-loaded glassy carbon electrode (5 mm in diameter, effective area of 0.19625 cm^2^) as the working electrode, and 0.1 M KOH as the electrolyte. The use of this alkaline medium is confined to the half-cell ORR measurements described in this section, because KOH at this concentration is the conventional electrolyte for benchmarking ORR electrocatalysts and therefore allows the obtained potentials and current densities to be directly compared with the values reported in the literature; in contrast, the Mg–air battery tests were carried out in 3.5 wt.% NaCl aqueous solution (see Section 2.2) to avoid anodic passivation of the metallic Mg anode. To facilitate the comparison of catalyst performance across different experimental systems, the potential was converted to the reversible hydrogen electrode (RHE) scale according to

E_RHE_ = E_Hg/HgO_ + 0.059 pH + 0.098 V (1)

All potentials reported in this work are referred to the RHE. Prior to the measurements, O_2_ or N_2_ was bubbled into the electrolyte for 30 min to achieve the corresponding gas-saturated state. For the transition-metal Mo-based catalysts in this work, the catalyst ink was prepared as follows: 15 mg of the catalyst and 3 mg of Ketjenblack were weighed, ground thoroughly in an agate mortar, and then transferred into a 5 mL vial, followed by the sequential addition of 250 μL of deionized water, 250 μL of anhydrous ethanol, and 70 μL of Nafion solution (10 wt.%). The resulting mixture was ultrasonicated for 1 h to achieve uniform dispersion, with occasional shaking during sonication to prevent catalyst agglomeration. Subsequently, 5 μL of the ink was pipetted onto the electrode surface, corresponding to a mass loading of approximately 0.8 mg cm^−2^, and the electrode was allowed to dry naturally in air before use.

Cyclic voltammetry (CV) and Linear sweep voltammetry (LSV) measurements were carried out on a CHI760E electrochemical workstation at room temperature, using a three-electrode system with 0.1 M KOH as the electrolyte. Prior to the CV measurements, N_2_ was purged for 30 min to reach saturation, the electrode was first activated by continuous scanning for 40 cycles at a scan rate of 50 mV s^−1^ and a rotation speed of 1600 rpm; subsequently, at the same scan rate and rotational speed, 19 cycles of CV curves were recorded within the potential range of −1 to 0.5 V under both N_2_-saturated and O_2_-saturated conditions, the corresponding RHE potential window was −0.132 to 1.368 V. LSV measurements were performed at a scan rate of 10 mV s^−1^ over a potential range of −0.9 to 0.2 V (corresponding to the RHE window of −0.032 to 1.068 V) at electrode rotation rates of 625, 900, 1225, 1600 and 2025 rpm. All polarization curves reported in this work were background-subtracted using the polarization curve measured in N_2_-saturated electrolyte as the background, thereby eliminating the influence of double-layer capacitive current and non-ORR side reactions on the current signal; the resulting current is the net oxygen reduction current. Meanwhile, the onset potential (E_onset_), half-wave potential (E_1/2_), and diffusion-limited current density (j_L_) derived from the LSV polarization curves of catalysts phosphidized at different temperatures at 1600 rpm were adopted as the metrics for evaluating the ORR catalytic activity. Open-circuit voltage (OCV), rate performance, and galvanostatic discharge tests of the Mg–air batteries were all carried out on a LAND battery testing system (Land CT2001A device) at room temperature. The OCV was measured at a sampling frequency of 5 Hz to record the stable potential difference between the positive and negative electrodes of the battery under open-circuit conditions for 1800 s. The rate performance was evaluated under a stepwise current protocol at current densities of 10, 20, 40, 50, 40, 20, and 10 mA cm^−2^, with each current step maintained for 1 h. Galvanostatic discharge measurements were conducted at constant current densities of 5 and 10 mA cm^−2^, during which the discharge voltage was recorded as a function of time to evaluate the discharge stability of the batteries.

## 3. Results and Discussion

**Figure 1:**
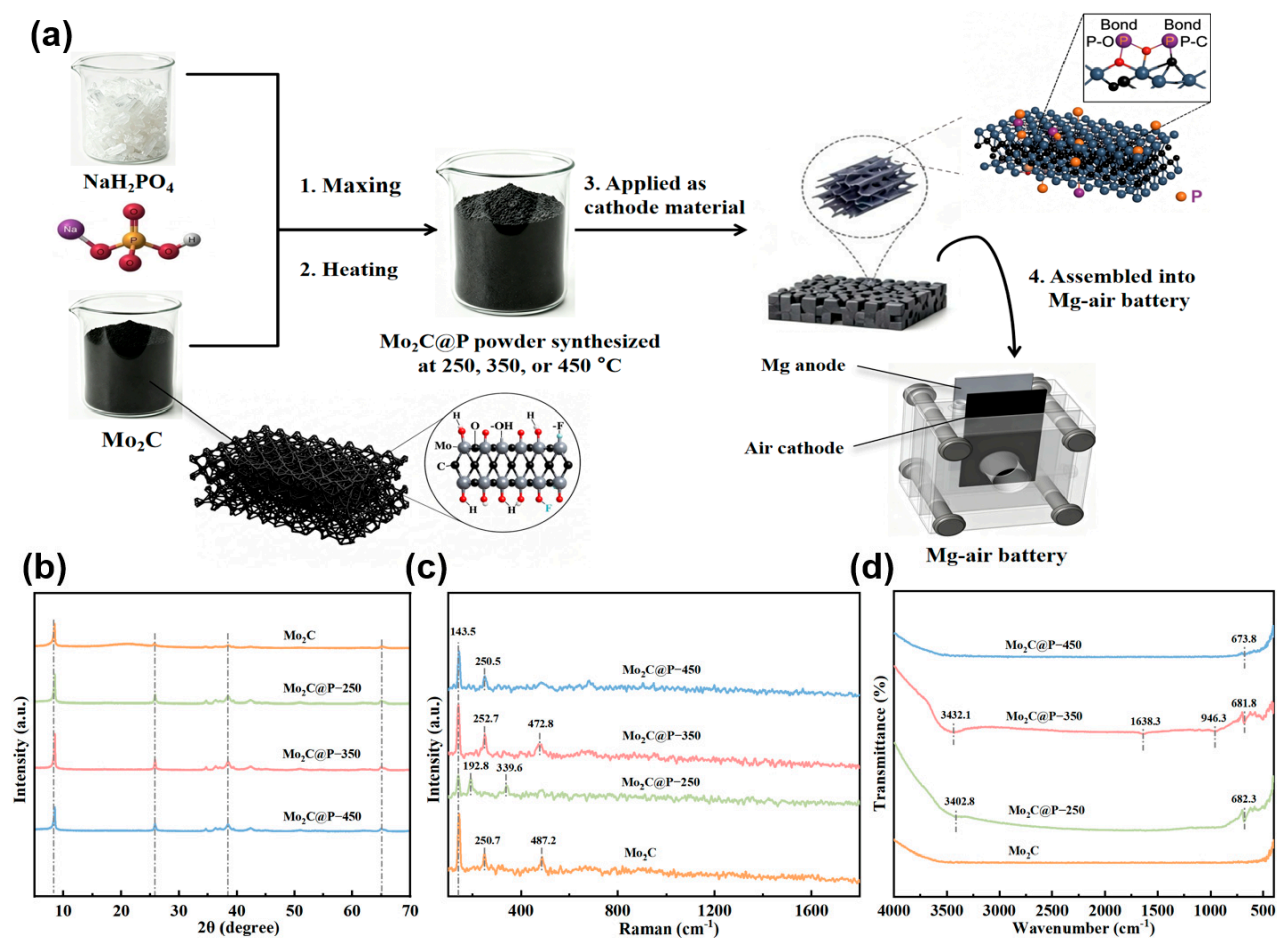
(**a**) Schematic illustration of the preparation procedure of Mo_2_C@P; (**b**) XRD patterns, (**c**) Raman spectra and (**d**) FT-IR spectra of Mo_2_C and Mo_2_C@P phosphidized at different temperatures.

As illustrated in Figure 1a, Mo_2_C@P catalysts were prepared by mechanically mixing Mo_2_C powder with sodium hypophosphite (NaH_2_PO_2_) at a mass ratio of 10:1, purging with N_2_ for 1 h, and then phosphidizing at 250, 350 and 450 °C (5 °C min^−1^, held for 2 h) under a nitrogen atmosphere, followed by natural cooling to room temperature, affording Mo_2_C@P–250, Mo_2_C@P–350, and Mo_2_C@P–450. Figure 1b presents the XRD patterns of pristine Mo_2_C and the Mo_2_C@P samples phosphidized at different temperatures. All four samples exhibit identical diffraction features: the peak at 2θ ≈ 8.5° corresponds to the (002) reflection of the layered Mo_2_C MXene, serving as a signature of the ordered stacking of MXene sheets; the strong peak at 2θ ≈ 38.5° and the peak at 65° are consistent with the characteristic reflections of β–Mo_2_C [15,24]; and the weak peak around 26° can be assigned to the (002) plane of the graphite-carbon, as also reported for Mo_2_C-based materials [24]. A few additional weak reflections are observed at 2θ ≈ 34.7°, 36.4°, 37.1°, 39.4°, 42.4°, 43.7°, and 45.1°, which are also present in the pristine Mo_2_C pattern and whose positions remain identical (within ±0.2°) across all four samples. These reflections can be assigned to the β–Mo_2_C phase, since they fall within the angular range of its characteristic peaks (34.35°, 38.98°, 39.39° and 52.12° for the (100), (002), (101) and (102) planes) [24]. Importantly, no new peak position appears after phosphidation at any of the three temperatures, which indicates that no diffraction signal from new crystalline phosphorus-containing phases such as molybdenum phosphides, oxides, or phosphates is detected. This conclusion is consistent with the very low phosphorus content expected for a surface-modification route, which is far below the detection limit of XRD for such a light element. The phosphidation process therefore does not damage the bulk crystal structure of the Mo_2_C MXene: phosphorus is not incorporated into the lattice to form new crystalline phases, but is instead introduced into the surface and near-surface regions in a low-content, amorphous form, with its content and crystallinity below the detection limit of XRD. This demonstrates that the mechanical mixing followed by programmed-temperature phosphidation adopted in this work is a mild surface-modification route, under which the layered framework of the Mo_2_C MXene remains intact after phosphidation, providing a stable structural foundation for the subsequent electrocatalytic reactions. As shown in Figure 1c, the two characteristic peaks of pristine Mo_2_C at 250.7 and 487.2 cm^−1^ correspond to the skeletal Mo–C vibration and the surface Mo–O species, respectively [25]. The low-frequency band at 143.5 cm^−1^ corresponds to the in–plane 2Eg mode of the Mo_2_C lattice [25]. These two Mo_2_C signals are retained in Mo_2_C@P–350 at 252.7 and 472.8 cm^−1^, respectively. The nearly unchanged Mo–C position indicates that the Mo_2_C framework is preserved. Phosphidation at 350 °C therefore proceeds without disrupting the framework while maintaining intrinsic surface oxygen-containing species, corresponding to a moderate surface chemical state. In contrast, the spectrum of Mo_2_C@P–250 is dominated by two new bands at 192.8 and 339.6 cm^−1^, which can be assigned to MoO_2_, whose characteristic bands are reported at approximately 192 and 340 cm^−1^, respectively [26]. Correspondingly, the skeletal Mo–C vibration at 250.7 cm^−1^ observed in the pristine Mo_2_C disappears completely in this sample, indicating that the surface Mo_2_C layer is oxidized rather than phosphidized at this temperature. This behavior originates from the insufficient amount of phosphine generated in situ: NaH_2_PO_2_ disproportionates on heating to release PH_3_ (2NaH_2_PO_2_ → Na_2_HPO_4_ + PH_3_↑) [27], which is the active species that reacts with the Mo_2_C surface to form the P–O and P–C linkages. Since the rapid decomposition of NaH_2_PO_2_ starts only above approximately 250 °C [27], the phosphine generated at this temperature is insufficient to convert the oxygen-containing terminations (–O, –OH, =O) that inherently cover the Mo_2_C surface [15]. The surface oxygen species therefore remain and oxidize the outermost Mo_2_C layer into MoO_2_, which accounts for both bands and for the disappearance of the skeletal Mo–C vibration. In Mo_2_C@P–450, the lattice and skeletal vibrations at 143.5 and 250.5 cm^−1^ are retained, whereas the signals of surface species in the 470–490 cm^−1^ region become weak and indistinct, indicating that excessive temperature causes a considerable loss of surface oxygen- and phosphorus-containing species. Moreover, no obvious D or G bands are observed near 1350 and 1580 cm^−1^ for any of the samples, confirming the absence of significant free amorphous or graphitic carbon, and that the carbon element exists mainly in the form of the Mo_2_C framework. In Figure 1d, Mo_2_C@P–350 simultaneously exhibits four types of characteristic absorptions at 3432.1, 1638.3, 946.3, and 681.8 cm^−1^, presenting the most complete surface chemical information among the four samples. The broad band at 3432.1 cm^−1^ and the absorption at 1638.3 cm^−1^ are assigned to the O–H stretching vibration of surface hydroxyl groups and adsorbed water and to the bending vibration of adsorbed H–O–H, respectively [28]. The peak at 946.3 cm^−1^ lies in the region of P–O stretching vibrations, where the phosphoryl and P–O–C groups of phosphorus-doped carbons are observed at about 950 and 1050 cm^−1^ [29], while the peak at 681.8 cm^−1^ corresponds to the P–C stretching vibration, whose frequency has been calculated to fall between 670 and 780 cm^−1^ for phosphorus bonded to carbon [28,30]. Together, they confirm that phosphorus is introduced onto the Mo_2_C surface in two bonding forms, P–O and P–C. It should be noted that no corresponding absorption is observed near 946 cm^−1^ for pristine Mo_2_C, which excludes the possibility that this peak originates from the Mo=O vibration of surface molybdenum oxide and further supports its assignment to P–O bonds. In contrast, Mo_2_C@P–250 exhibits a markedly weaker P–O absorption at 946.3 cm^−1^, although the P–C absorption, shifted to 682.3 cm^−1^, remains visible, indicating that the formation of P–O linkages is incomplete at 250 °C owing to limited decomposition of NaH_2_PO_2_. Mo_2_C@P–450 retains only a weak absorption at 673.8 cm^−1^, while the P–O band at 946.3 cm^−1^ and the O–H band near 3400 cm^−1^ are almost no longer resolved. The loss of the O–H band is consistent with the thermal behavior of Mo_2_CT_x_, in which physically adsorbed water is removed below about 200 °C and the –OH/–O terminations are progressively lost between 200 and 500 °C [31]. The disappearance of the P–O band, together with the markedly weakening of the P–C band, shows that the P–O configuration formed at lower temperature is not retained at 450 °C. Excessive temperature therefore causes a substantial loss of surface hydroxyl groups and of the phosphorus-containing surface species, resulting in a significant decline in surface functionalization. Among the three phosphidation temperatures, 350 °C is the condition under which the P–O and P–C linkages and hydroxyl groups are most completely retained.

**Figure 2:**
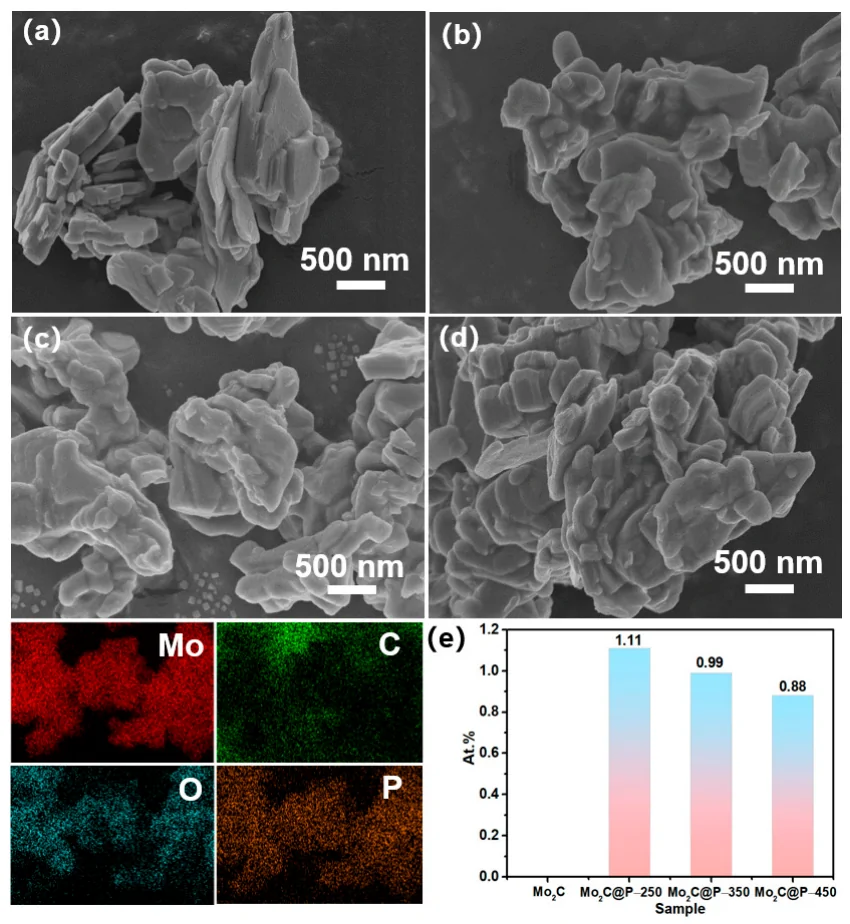
SEM images of (**a**) pristine Mo_2_C and (**b**–**d**) Mo_2_C@P samples after phosphidation at 250, 350, and 450 °C, respectively; (**e**) atomic percentage of P in pristine Mo_2_C and the Mo_2_C@P samples phosphidized at different temperatures.

Scanning electron microscopy (SEM) was employed to characterize the evolution of sample microstructure at different phosphidation temperatures. Figure 2a–d present the SEM images of pristine Mo_2_C and Mo_2_C samples phosphidized at 250, 350, and 450 °C, respectively. Pristine Mo_2_C (Figure 2a) exhibits a stacked lamellar structure with sharp edges and flat crystalline facets. After phosphidation at 250 °C (Figure 2b), the lamellar surface becomes slightly roughened, and the sharp edges are passivated, while the overall lamellar morphology is largely retained. As the phosphidation temperature is raised to 350 °C (Figure 2c), the lamellar structure undergoes further reconstruction: uniform particle-like modification is formed on the surface, and the originally sharp sheet boundaries largely disappear. A further increase in the phosphidation temperature to 450 °C (Figure 2d) leads to pronounced agglomeration and severe distortion of the stacked lamellar structure. The elemental mapping of the sample phosphidized at 350 °C (inset of Figure 2c) reveals that Mo, C, O, and P are uniformly distributed over the entire scanned region, confirming the successful incorporation of phosphorus into the Mo2C matrix. Figure 2e summarizes the atomic percentages of P in all samples: with increasing phosphidation temperature, the P atomic ratio decreases progressively, reaching a maximum of 1.11 at.% at 250 °C, with values of 0.99 at.% and 0.88 at.% for the samples phosphidized at 350 and 450 °C, respectively. This maximum does not imply more extensive phosphidation at 250 °C, because Energy–dispersive X–ray spectroscopy (EDX) quantifies phosphorus irrespective of its chemical state and thus also counts phosphorus from unreacted NaH_2_PO_2_. In gas–solid phosphidation, NaH_2_PO_2_ must first decompose to release PH_3_, and this rapid decomposition begins only above approximately 250 °C [27], so that a substantial part of the precursor remains unreacted at this temperature; a large amount of unreacted NaH_2_PO_2_ has been directly reported after phosphidation at 473 K [32]. Above 250 °C, where NaH_2_PO_2_ decomposes rapidly, the lower P atomic percentages at 450 °C are attributed to the thermal desorption of the phosphorus-containing surface species, which accompanies the progressive loss of the –OH/–O terminations of Mo_2_CT_x_ between 200 and 500 °C [31], since the grafted phosphorus is bound to the outermost surface through these terminations [33]. The concurrent loss of the O–H band in the FTIR spectra (Figure 1d) is consistent with this picture. In addition, the high temperature induces close agglomeration of particles, which impedes diffusion of the phosphorus precursor and further reduces the phosphorus content retained in the materials.

**Figure 3:**
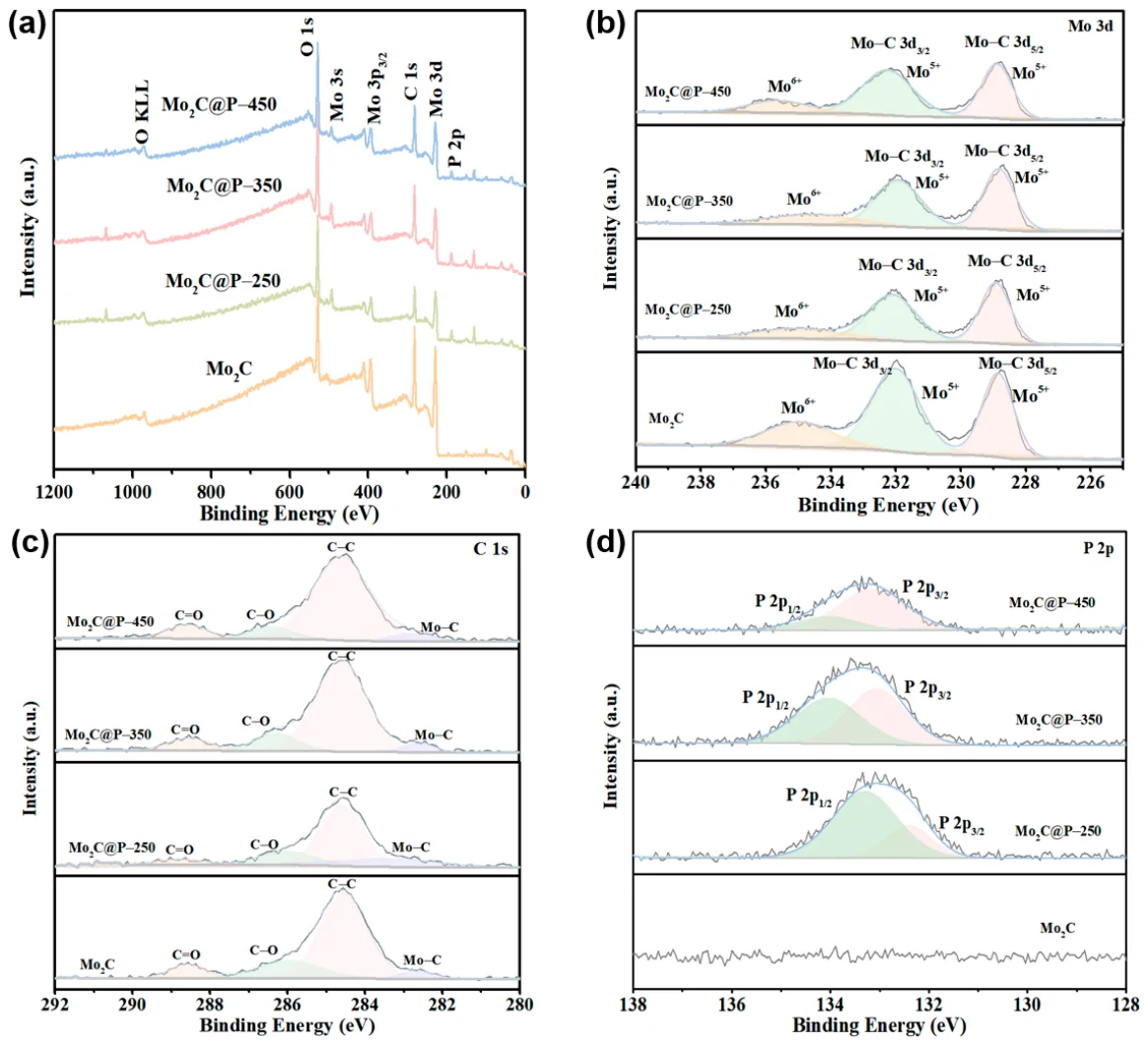
XPS spectra of pristine Mo_2_C and the Mo_2_C@P samples phosphidized at different temperatures: (**a**) survey spectra; high-resolution spectra of (**b**) Mo 3d, (**c**) C 1s, and (**d**) P 2p.

XPS was employed to analyze the surface elemental composition and electronic structure of pristine Mo_2_C and the samples phosphidized at 250, 350, and 450 °C. Figure 3a exhibits the survey spectra of all samples, in which the characteristic signals of Mo, C, and O are observed. The phosphidized samples additionally exhibit the characteristic peak of P, confirming the successful incorporation of phosphorus into the Mo_2_C matrix, whereas no discernible P signal is detected for pristine Mo_2_C. Quantitative XPS analysis reveals that the surface P atomic content decreases progressively with increasing phosphidation temperature: it reaches 8.66 at.% for the sample phosphidized at 250 °C, decreases to 6.34 at.% at 350 °C, and further drops to 5.14 at.% at 450 °C, demonstrating that elevated phosphidation temperatures induce the desorption and loss of phosphorus species. It is worth noting that the EDX and XPS values should not be compared directly, since the two techniques quantify different depths in the same sample. The characteristic X-rays used for EDX are excited within an electron interaction volume of the order of 1 μm^3^ [34], which is far deeper than the outermost ~1–10 nm from which XPS derives its information by photoelectron escape. A similar difference has been reported for functionalized graphene nanoplatelets, where the O atomic percentage measured by XPS was about three times the EDX value, which was attributed to the functionalization being concentrated within the first 10 nm [35]. In the present case, the phosphorus introduced by phosphidation is concentrated in the near-surface region of the stacked Mo_2_C lamellae rather than uniformly distributed through them. This is consistent with the grafting route: with NaH_2_PO_2_ as the phosphorus source, the phosphorus incorporated into Ti_3_C_2_T_x_ MXene is bound to the outermost surface through oxygen terminations rather than dispersed into the lattice [33]. Therefore, XPS probes the phosphorus-rich outer layer within a depth of about 10 nm, whereas the EDX value is averaged over a much larger volume, so that the P atomic percentage determined by XPS (5.14–8.66 at%) is considerably higher than the EDX value (0.88–1.11 at%). Figure 3b reveals the high-resolution Mo 3d spectra of all samples. The Mo 3d spectrum of pristine Mo_2_C can be deconvoluted into two components corresponding to Mo–C and Mo^6+^. After phosphidation, the characteristic Mo–C peak is still retained, indicating that the treatment does not damage the intrinsic crystal framework of Mo_2_C; meanwhile, the relative proportion of Mo^6+^ changes, which originates from the rearrangement of the electronic structure of Mo sites induced by surface phosphidation modification. As the phosphidation temperature increases, the binding energies of Mo species shift slightly, reflecting the interaction formed between P and Mo and the resulting redistribution of the surface electron cloud around Mo atoms. Figure 3c depicts the high-resolution C 1s spectra, which can be deconvoluted into four characteristic components of Mo–C, C–C, C–O, and C=O. The C–C signal originates from the carbon substrate on the material surface, while C–O and C=O are attributed to the oxidized carbon species derived from oxygen adsorbed on the sample surface. Variations in phosphidation temperature have little influence on the positions of the characteristic peaks of the carbon framework, proving that this phosphidation process exerts a limited effect on the structure of the carbon matrix. Figure 3d presents the high-resolution P 2p spectra. No P 2p signal is observed for pristine Mo_2_C, whereas the samples phosphidized at 250, 350, and 450 °C can all be fitted with characteristic P 2p1/2 and P 2p3/2 peaks, confirming successful doping of phosphorus species. For Mo_2_C@P-350, the fitted binding energies are shifted to higher values relative to Mo_2_C@P-250, which reflects a higher electron deficiency of the phosphorus, i.e., a larger extent of P–O bonding to the surface oxygen terminations at this temperature; the fitted values fall in the range reported for the oxygen-containing phosphorus species of phosphorus-doped carbon layers [36] and agree with the P–O species observed for phosphidated MXenes [33]. This is consistent with the increase in the P–O band in the FTIR spectra between 250 and 350 °C (Figure 1d). As the phosphidation temperature increases, the intensity of the P 2p peaks weakens progressively, consistent with the trend in P atomic percentage obtained from XPS quantification, further proving that phosphorus species volatilize and desorb at elevated temperatures, leading to a continuous decrease in surface phosphorus content.

In summary, the phosphidation temperature can simultaneously regulate the microstructure of Mo_2_C, the surface phosphorus loading, and the electronic structure of Mo sites. A lower phosphidation temperature (250 °C) affords a higher surface P atomic content; phosphidation at 350 °C yields a rough particle-like surface with uniformly distributed phosphorus in the matrix; further increasing the temperature to 450 °C not only causes the loss of phosphorus species but also induces particle agglomeration.

**Figure 4:**
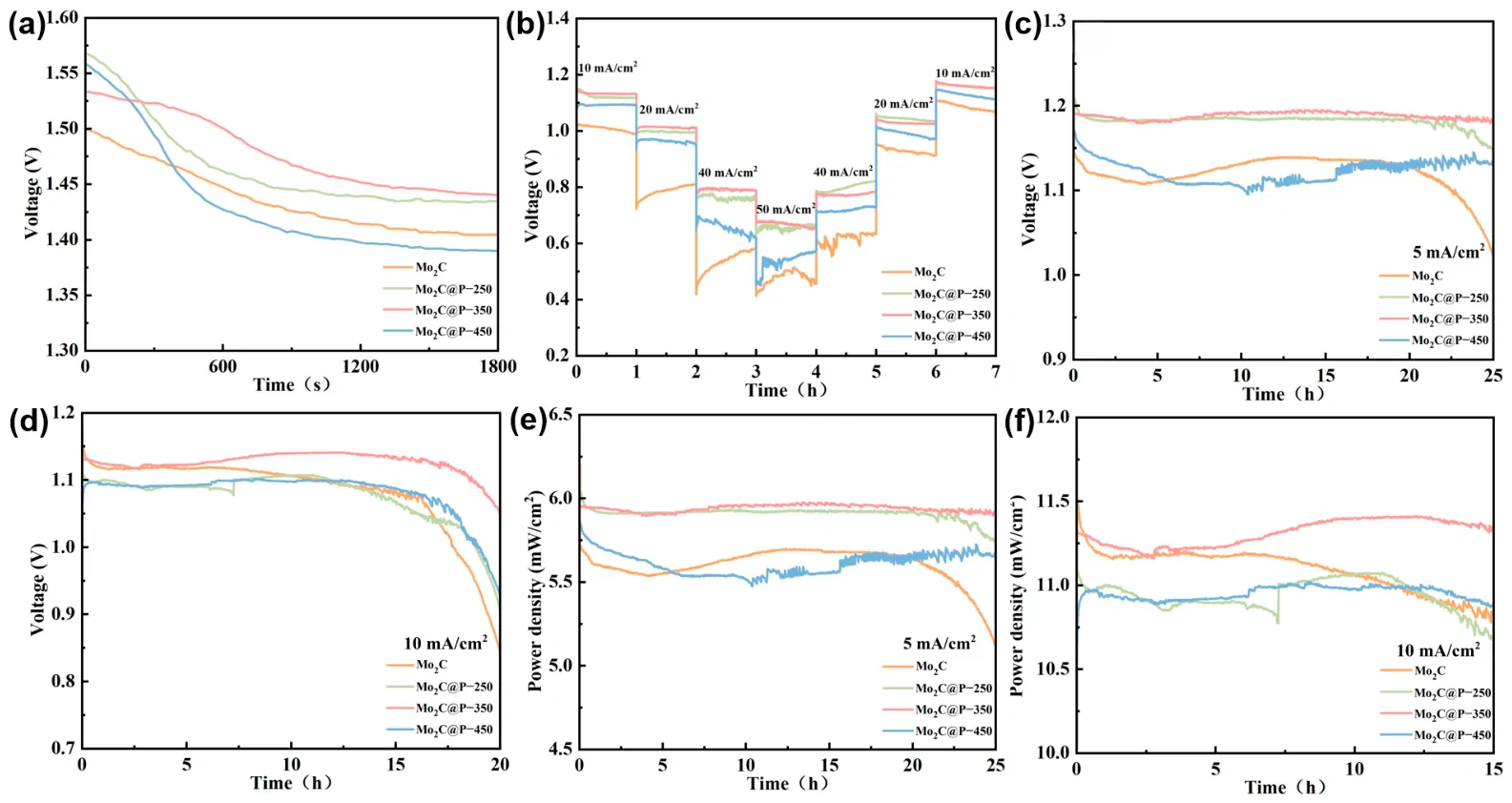
Electrochemical performance of Mg–air batteries assembled with pristine Mo_2_C and the Mo_2_C@P samples phosphidized at different temperatures: (**a**) open-circuit voltage curves; (**b**) rate performance at stepped current densities of 10, 20, 40, and 50 mA cm^−2^; galvanostatic discharge curves at current densities of (**c**) 5 mA cm^−2^ and (**d**) 10 mA cm^−2^; and power density curves at current densities of (**e**) 5 mA cm^−2^ and (**f**) 10 mA cm^−2^.

As shown in Figure 4a, the initial open-circuit voltages of the batteries range from 1.50 to 1.58 V, among which the Mo_2_C@P–250 battery is the highest (~1.57 V) and the pristine Mo_2_C battery is the lowest (~1.50 V). During the 1800 s standing process, the voltages of all batteries decay slowly, but the decay amplitudes differ significantly: the Mo_2_C@P–350 battery decays by only about 0.09 V and still maintains ~1.44 V at the end of 1800 s, which is the highest among all batteries; the final voltages of the Mo_2_C@P–250 and Mo_2_C batteries are ~1.43 V and 1.40 V, respectively; the Mo_2_C@P–450 battery shows the most pronounced decay, with a final voltage of approximately 1.39 V. The magnitude of the open-circuit voltage can approximately reflect the thermodynamic electromotive force level of the battery, whereas the OCV decay rate during standing reflects the stability of the electrode–electrolyte interface, because at open circuit the electrons released by the Mg anode are diverted into the electrolyte, causing a parasitic self-discharge and the formation of a passivation layer at the anode–electrolyte interface [5,37]. The extent of this self-discharge further depends on the ability of the air cathode to suppress the competing hydrogen evolution reaction [18], so that the different decay amplitudes of the four samples reflect the different interfacial states of their catalytic layers. Although the initial voltage of the Mo_2_C@P–350 battery is not the highest, its decay is the slowest and its final voltage is the highest, indicating that the electrode–electrolyte interface of this battery is the most stable under standing conditions, with low degrees of self-discharge and interfacial side reactions; in contrast, the rapid decay of the Mo_2_C@P-450 battery is associated with its pronounced surface agglomeration and non-uniform interfacial reactions. Figure 4b presents the rate discharge curves of the batteries under a stepwise protocol of 10–20–40–50–40–20–10 mA cm^−2^. At the initial stage of 10 mA cm^−2^, the Mo_2_C@P–350 and Mo_2_C@P–250 batteries deliver the highest discharge voltages (~1.14 V), followed by Mo_2_C@P–450 (~1.10 V), with pristine Mo_2_C being the lowest (~1.02 V). As the current density increases, the discharge voltages of all batteries decrease stepwise owing to increased polarization; at the high current density of 50 mA cm^−2^, Mo_2_C@P–350 still maintains approximately 0.68 V, whereas pristine Mo_2_C has dropped to approximately 0.45 V with obvious fluctuations. When the current density decreases stepwise from 50 mA cm^−2^ back to 10 mA cm^−2^, the discharge voltages of all batteries recover accordingly and are generally higher than their initial levels at the same current densities. This phenomenon originates from the further infiltration of the electrolyte into the electrode pores during the high-current stage, the in situ activation of the catalyst [17], and the unblocking of mass-transport channels, which reduce the system polarization, since the oxygen reduction takes place in the electrolyte-filled pores of the porous air cathode at the three-phase interface [37]. Among them, Mo_2_C@P-350 shows the highest voltage, recovering to approximately 1.19 V. By contrast, Mo_2_C@P-450 suffers from aggravated polarization in the high-current region, with poorer voltage stability and weaker reversible recovery capability, which is significantly lower than those of the other phosphidized samples and only slightly better than that of the unphosphidized pristine Mo_2_C. The above comparative analysis demonstrates that the Mo_2_C@P-350 catalytic layer maintains its structural integrity after high-current impact and possesses good rate reversibility. Figure 4c,d show the galvanostatic discharge curves of the batteries at 5 and 10 mA cm^−2^. At the low current density of 5 mA cm^−2^, the Mo_2_C@P-350 battery maintains a stable voltage plateau of approximately 1.19 V throughout the 25 h test, which is overall superior to that of Mo_2_C@P–250; the Mo_2_C@P–250 battery shows a comparable voltage in the early stage (~1.18 V), which, however, begins to decline to 1.15 V after 20 h. The voltage of the Mo_2_C@P–450 battery decreases from 1.15 V to 1.11 V and then rises slightly, but its curve exhibits dense small-amplitude oscillations, indicating continuous slight voltage fluctuations. The pristine Mo_2_C battery delivers a voltage of ~1.14 V, but with a large fluctuation amplitude and poor stability during discharge, and undergoes a sudden voltage drop after 19 h, falling to ~1.02 V at 25 h. At the higher current density of 10 mA cm^−2^, the differences among the batteries are further amplified: although the initial voltage of the pristine Mo_2_C battery is close to that of Mo_2_C@P–350 (~1.13 V), its voltage drops sharply after 16 h to only 0.85 V at the end; the Mo_2_C@P–250 and Mo_2_C@P–450 batteries show similar voltages within the first 12 h, followed by gradual decay, reaching final voltages of 0.92 V and 0.93 V, respectively; the Mo_2_C@P–350 battery maintains 1.14 V for 16 h, which is the highest overall, with the smallest voltage decay, and its final voltage reaches 1.05 V. The power density of the batteries is presented in Figure 4e,f. At 5 mA cm^−2^, the Mo_2_C@P–350 battery delivers approximately 5.95 mW cm^−2^ and maintains this value throughout the 25 h test, whereas the pristine Mo_2_C battery delivers about 5.7 mW cm^−2^ and decays continuously after 20 h; at 10 mA cm^−2^, Mo_2_C@P–350 delivers approximately 11.4 mW cm^−2^, the highest and most stable value among the four samples.

Based on the above results, the Mg–air battery assembled with Mo_2_C@P–350 exhibits the best performance in terms of discharge voltage level, discharge stability, and power density.

**Figure 5:**
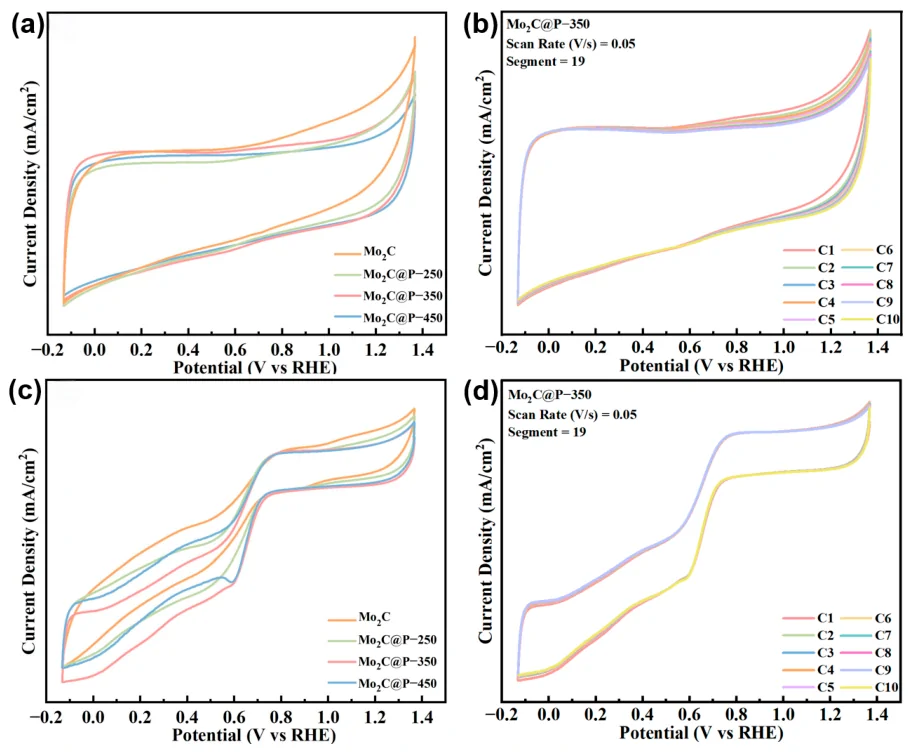
CV curves of pristine Mo_2_C and the Mo_2_C@P samples phosphidized at different temperatures: (**a**) in N_2_-saturated electrolyte; (**b**) for Mo_2_C@P-350 recorded over 19 consecutive segments in N_2_-saturated electrolyte; (**c**) in O_2_-saturated electrolyte; (**d**) for Mo_2_C@P-350 recorded over 19 consecutive segments in O_2_-saturated electrolyte.

As shown in Figure 5a, the CV curves recorded under N_2_–saturated conditions all exhibit a quasi-rectangular shape without obvious redox peaks, showing typical electric double-layer capacitive behavior, and the four samples differ only slightly in capacitive current, indicating comparable double-layer capacitances. Figure 5c shows that the CV curves measured under O_2_-saturated conditions all display distinct reduction peaks, demonstrating that these peaks arise from O_2_ reduction, namely the oxygen reduction reaction (ORR). In addition, CV curves of catalysts phosphidized at different temperatures differ from one another; a more positive reduction peak position, together with a larger current density, indicates higher ORR catalytic activity of the catalyst. The reduction peak potentials of Mo_2_C@P–250, Mo_2_C@P–350, and Mo_2_C@P–450 are 0.504, 0.594, and 0.586 V, respectively, among which Mo_2_C@P–350 exhibits the most positive reduction peak potential and the largest current density, preliminarily indicating its highest ORR catalytic activity. Meanwhile, as shown in Figure 5b,d, the CV curves of Mo_2_C@P–350 recorded over 19 consecutive segments (C1–C10) under N_2_-saturated and O_2_-saturated conditions, respectively, are essentially overlapped without obvious fluctuation differences, demonstrating the stability of the test system and the excellent reproducibility of the data.

**Figure 6:**
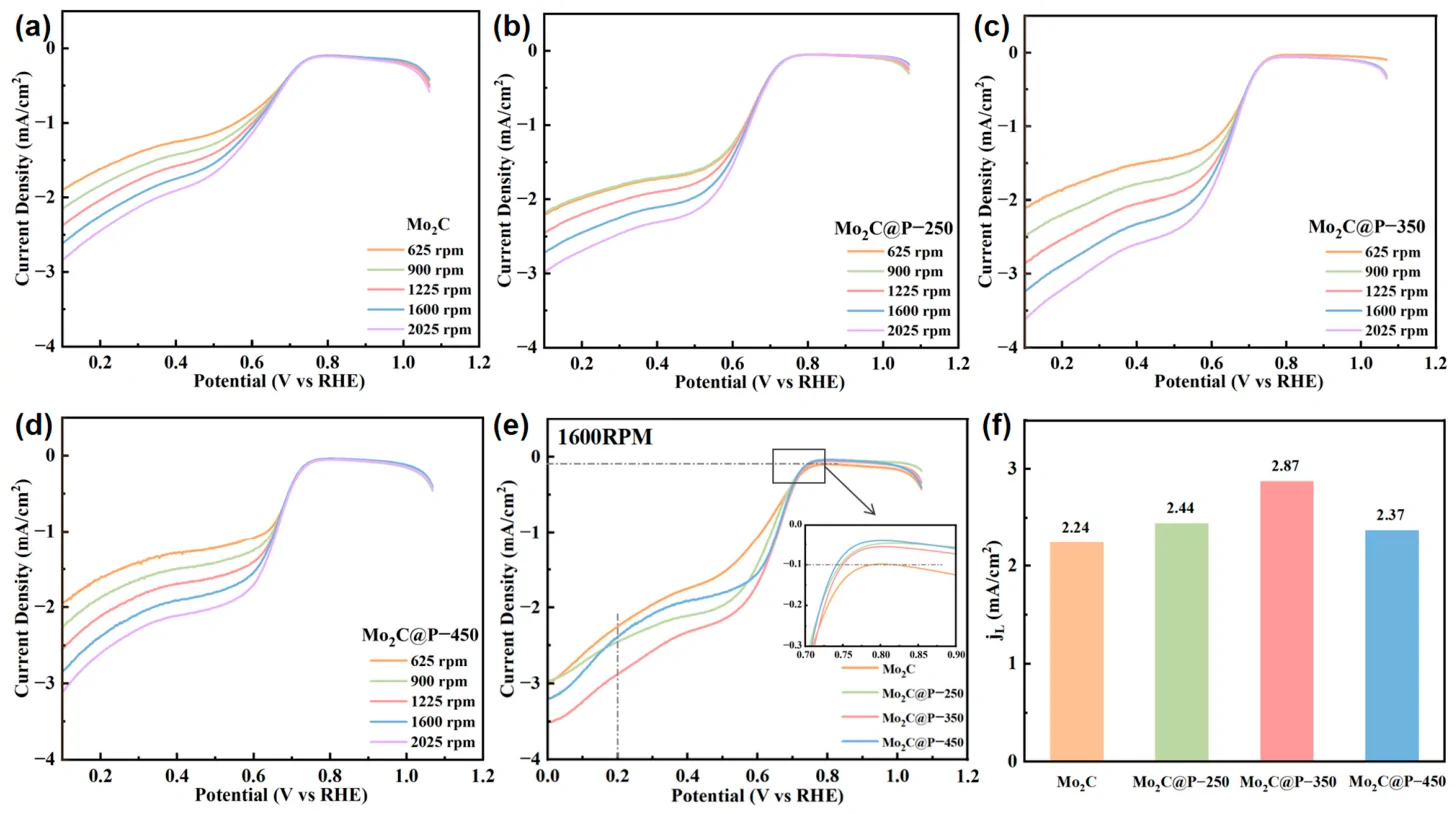
LSV curves of pristine Mo_2_C and the Mo_2_C@P samples phosphidized at different temperatures: LSV curves recorded at rotation rates of 625, 900, 1225, 1600, and 2025 rpm for (**a**) Mo_2_C and (**b**–**d**) Mo_2_C@P–250, Mo_2_C@P–350, and Mo_2_C@P–450, respectively; (**e**) LSV curves at a rotation rate of 1600 rpm, with the inset showing an enlarged view of the onset potential region; (**f**) diffusion-limited current densities (j_L_) of all samples at 1600 rpm.

In Figure 6a–d, the LSV curves of pristine Mo_2_C and the Mo_2_C@P samples phosphidized at different temperatures demonstrate that the continuous increase in rotation rate drives the dissolved oxygen in the electrolyte to be transported more rapidly to the three-phase reaction interface at the catalyst surface, while the reduction products generated by the reaction can desorb more quickly from the electrode surface and diffuse into the electrolyte, thereby reducing the mass-transfer resistance; consequently, the diffusion-limited current density increases continuously, which is consistent with the diffusion-controlled characteristics of the oxygen reduction process on a rotating disk electrode. As annotated in Figure 6e, in this work the onset potential (E_onset_) of the catalysts is defined as the potential corresponding to 0.1 mA cm^−2^, the half-wave potential (E_1/2_) as the potential corresponding to half of the diffusion-limited current density (j_L_), and the diffusion-limited current density (j_L_) as the current density at 0.2 V; the results are summarized in Table 1. As shown in Table 1 and Figure 6f, the Mo_2_C@P-250, Mo_2_C@P-350, and Mo_2_C@P-450 catalysts possess similar onset and half-wave potentials, whereas Mo_2_C@P-350 exhibits the largest diffusion-limited current density. As the phosphidation temperature increases from 250 to 350 °C, E_1/2_ shifts positively step by step and j_L_ increases accordingly; when the phosphidation temperature further rises to 450 °C, j_L_ decreases to 2.37 mA cm^−2^. Although the E_1/2_ of this sample (0.64 V) is slightly more positive than those of the other samples, the decline in limiting current indicates a reduction in the number of effective active sites in the high-temperature-phosphidized sample. This is attributed to the pronounced agglomeration and distortion of the stacked lamellar structure observed by SEM, which covers part of the surface active sites, together with the depletion of surface oxygen- and phosphorus-containing functional groups evidenced by weakened P–O and P–C signals in the FTIR and Raman spectra. No crystalline phosphorus-containing secondary phase is involved, since no new diffraction signal is detected for this sample within the detection limit of XRD, and the Mo_2_C reflection intensities fall back to a level close to that of the pristine sample.

In summary, the ORR catalytic activity of Mo_2_C@P exhibits a volcano-type relationship with the phosphidation temperature, and 350 °C is the optimal phosphidation temperature. The P–O/P–C bonds and surface oxygen-containing functional groups introduced by moderate phosphidation are beneficial for optimizing the adsorption and desorption behavior of oxygen intermediates and enhancing the reaction kinetics; however, at excessively high temperatures, the agglomeration of the particles and the depletion of the surface functional groups offset the above benefits.

## 4. Conclusions

Overall, this work demonstrates that the long-standing kinetic limitation of the air cathode in Mg–air batteries can be effectively alleviated by unlocking the ORR activity of Mo_2_C MXene through a facile temperature-programmed phosphidation route with sodium hypophosphite as an inexpensive phosphorus source. The phosphidation temperature acts as a readily tunable descriptor of the catalyst: moderate phosphidation grafts phosphorus onto the surface oxygen terminations of the layered Mo_2_C framework through P–O and P–C linkages while preserving the framework and the native surface Mo–O species, whereas excessive treatment depletes the active phosphorus species and distorts the stacked lamellar structure, so that the phosphidation temperature has to be optimized rather than maximized. Correspondingly, ORR activity shows a volcano-type dependence on phosphidation temperature, with Mo_2_C@P–350 delivering the highest half-wave potential (0.62 V) and limiting diffusion current density (2.87 mA cm^−2^) in the series, compared with 0.59 V and 2.24 mA cm^−2^ for pristine Mo_2_C. As the air cathode of a Mg–air battery in 3.5 wt.% NaCl, it affords an open-circuit voltage of approximately 1.5 V with the smallest standing decay, stable discharge plateaus of approximately 1.19 V at 5 mA cm^−2^ for 25 h and 1.13 V at 10 mA cm^−2^ for 20 h, and power densities of approximately 5.95 and 11.4 mW cm^−2^.

Taken together, this study not only validates an MXene-based cathode beyond half-cell demonstrations in a practical Mg–air battery configuration but also establishes a generalizable design principle—decoupling framework stability from surface reactivity through mild, temperature-programmed heteroatom incorporation—that can be extended to other MXene compositions and heteroatom systems. When combined with electrolyte optimization and anode protection, such surface-engineered carbide catalysts are expected to help close the gap between the theoretical and practical energy density of low-cost, dendrite-free multivalent metal–air batteries on their way toward grid-scale and flexible energy storage. It should be noted that all the structural and spectroscopic data reported in this work were collected from the as-prepared catalysts. Stability of the catalyst during long-term discharge, which requires post-discharge characterization of the air electrode, has not been examined in this work and will be addressed in a follow-up study.

## Figures and Tables

**Table 1:** E_onset_, E_1/2_, and j_L_ of different catalysts at a rotation rate of 1600 rpm.

Sample	E_onset_/V (vs. RHE)	E_1/2_/V (vs. RHE)	j_L_/mA·cm^−2^
Mo_2_C	0.78	0.59	2.24
Mo_2_C@P-250	0.75	0.62	2.44
Mo_2_C@P-350	0.75	0.62	2.87
Mo_2_C@P-450	0.74	0.64	2.37

## Data Availability

The original contributions presented in this study are included in the article. Further inquiries can be directed to the corresponding authors.
